# Supplementary material for: Primary care provider uptake of intensive behavioral therapy for obesity in Medicare patients, 2013–2019
Source: PLoS One. 2023 Mar 24;18(3):e0266217. doi: 10.1371/journal.pone.0266217 (PMC10038247; doi:10.1371/journal.pone.0266217)
Supplement: S1 File — (DOCX) [file pone.0266217.s001.docx]

**Supplemental materials**

In addition to classifying providers as we did in the manuscript (early, late, always and never adopters), we also summarize the IBT uptake patterns among eligible primary care providers based on the total number of years they served at least 10 Medicare beneficiaries. A provider is a “rare user” if they used IBT for <50% of the years (1-3 years) or a “frequent user” if they used IBT for >50% (4-7 years). Table S1 shows the provider characteristics by this classification and Table S2 shows the multinomial logistic regression estimates.

The characteristics of providers who rarely used IBT (1-3 years out of 7 years) were similar to the late adopters in Table 1 and the frequent users (4-7 years) combined the characteristics of early adopters and always users.

The odds ratios (ORs) of the multinomial logistic regression with the three uptake categories in Table S2 were similar to those in Table 2. Again, the ORs for rare users were almost the same as the later adopters and the ORs for frequent users were in between the ORs for early adopters and always users (except for the Southwest region).

**Table S1** Provider characteristics by intensive behavioral therapy (IBT) uptake patterns in three categories (N=537,754) from 2013 to 2019

|  | **Never** | **Rare (1-3 years)** | **Frequent (4-7 years)** |
| --- | --- | --- | --- |
|  | N=531,587 | N=4,851 | N=1,316 |
| Gender |  |  |  |
| Male | 206528 (38.9%) | 2749 (56.7%) | 837 (63.6%) |
| Female | 325059 (61.1%) | 2102 (43.3%) | 479 (36.4%) |
| Region |  |  |  |
| Midwest | 128375 (24.1%) | 742 (15.3%) | 164 (12.5%) |
| Northeast | 109266 (20.6%) | 1186 (24.4%) | 401 (30.5%) |
| South | 193229 (36.3%) | 2084 (43.0%) | 567 (43.1%) |
| West | 100717 (18.9%) | 839 (17.3%) | 184 (14.0%) |
| Provider Type |  |  |  |
| Family Practice | 107270 (20.2%) | 1751 (36.1%) | 504 (38.3%) |
| General Practice | 7428 (1.4%) | 118 (2.4%) | 26 (2.0%) |
| Internal Medicine | 132094 (24.8%) | 2110 (43.5%) | 715 (54.3%) |
| Nurse Practitioner | 180882 (34.0%) | 651 (13.4%) | 55 (4.2%) |
| Physician Assistant | 103913 (19.5%) | 221 (4.6%) | 16 (1.2%) |
| Median annual number of unique Medicare beneficiaries | 108 | 268 | 344 |
| Median annual number of services/encounters per year | 264 | 1690 | 3096 |
| Median annual number of all procedure codes | 16 | 44 | 58 |
| Median annual submitted charge amount | 43955 | 173387 | 296086 |
| Percent of beneficiaries with obesity related chronic conditions ‡ | | | |
| Hypertension | 68.0% | 69.7% | 70.4% |
| Diabetes | 38.2% | 38.8% | 39.8% |
| Hyperlipidemia | 57.1% | 61.0% | 63.6% |

‡ Percentages based on providers with available data from Provider Summary Tables.

**Table S2** **Multinomial logistic regression of IBT update patterns in three categories and provider characteristics and patient composition.**

|  | **Never** | **Rare （1-3 years）** | | **Frequent (4-7 years)** | |
| --- | --- | --- | --- | --- | --- |
|  |  | **OR** | **(95% CI)** | **OR** | **(95% CI)** |
| Male | 38.9% | Ref |  | Ref |  |
| Female | 61.1% | 0.68 | (0.64,0.72) | 0.58 | (0.51,0.65) |
| Midwest | 24.1% | Ref |  | Ref |  |
| Northeast | 20.6% | 1.69 | (1.54,1.86) | 2.35 | (1.95,2.82) |
| South | 36.3% | 1.67 | (1.54,1.82) | 1.95 | (1.63,2.32) |
| West | 18.9% | 1.49 | (1.35,1.65) | 1.49 | (1.21,1.84) |
| Other PCPs† | 75.2% | Ref |  | Ref |  |
| Internal medicine | 24.8% | 1.54 | (1.44,1.64) | 2.06 | (1.82,2.32) |
| # Medicare beneficiaries /100 | 1.8 | 1.08 | (1.07,1.09) | 1.08 | (1.07,1.09) |
| # services encounters /100 | 9.4 | 1.00 | (1.00,1.00) | 1.00 | (1.00,1.00) |
| $ submitted charges /10,000 | 11.2 | 1.04 | (1.03,1.04) | 1.05 | (1.04,1.05) |
| % patients w/ hypertension /10 ‡ | 6.8 | 0.94 | (0.90,0.99) | 0.81 | (0.74,0.89) |
| % patients w/ diabetes /10 ‡ | 3.8 | 0.92 | (0.90,0.95) | 0.92 | (0.88,0.97) |
| % patients w/ hyperlipidemia patients /10 ‡ | 5.7 | 1.28 | (1.24,1.32) | 1.62 | (1.51,1.72) |

† Other PCPs include family practice, general practice, nurse practitioner, and physician assistant.

‡ Missing data were imputed by the mean value. The percentages of missing data for hypertension, diabetes, and hyperlipidemia were 6.7%, 18.7%, and 11.2%.
