# Supplementary material for: Primary care provider uptake of intensive behavioral therapy for obesity in Medicare patients, 2013–2019
Source: PLoS One. 2023 Mar 24;18(3):e0266217. doi: 10.1371/journal.pone.0266217 (PMC10038247; doi:10.1371/journal.pone.0266217)
Supplement: Attachment — Submitted filename: Response to Reviewers.docx [file pone.0266217.s003.docx]

Thank you for the helpful comments by the academic editor and reviewers and for the opportunity to revise the manuscript. At the time of revision, we found the 2020 (latest) data for the Medicare Fee-for-service Provider Utilization and Payment data for Physician and Other Supplier Public Use File as well as the Provider Summary Tables from CMS became available. To provide more up to date information, we took the opportunity to add the year 2020 to the manuscript, hence the minor change in the title of the manuscript.

Below we address each reviewer comment. All page numbers referenced in this letter are those of the revised manuscript with track-changes. Blue font types are our response and brown font types are revised texts.

ACADEMIC EDITOR:

Remember Aim/Objective should be sound stated scientifically sounded and conclude accordingly: This:

"The purpose of this paper is to improve our understanding of the underutilization as well as the pattern of utilization of IBT for obesity services among PCPs serving the Medicare population across the U.S" does not give a clear idea of what is expected to be done.

Thank you for the comment. We clarified the objective by adding the following on p.4:

“The purpose of this paper is to examine the pattern of utilization of IBT for obesity services among PCPs serving the Medicare fee-for-service population across the U.S. to identify characteristics of providers who provided the services early, late, or never after the activation of the codes. Our analysis of provider characteristics complements and adds to our understanding of the uptake of IBT services by beneficiary characteristics [13,14].”

Please also provide de STROBE Checklist for your submission.

The Checklist is provided as a supplemental document.

Reviewer #1: The manuscript reports a description of data from publicly available sources, about use and implementation of a funded service directed to Medicare-beneficiaries with obesity. The study adds a new piece to the puzzle of understanding why the use of this service is low. Below I provide a few more general concerns with the analyses and how the manuscript is written, as well as minor comments to the text.

GENERAL CONCERNS
First, the tables referred to in the text were not available in the files I was sent (tables 1 and 2).

Tables were uploaded separately per PLOS ONE requirements but have now been added back into the paper.

Can you provide a more general discussion about how to interpret and understand the findings from this study outside of the fact that implementation is low? You found characteristics that identified service providers more or less likely to provide the service, but I can’t see any discussion about why the distribution of characteristics looks as it does or how this can be used to improve its use among those who are already eligible to provide it. Can you for example explain why female providers were less likely to take up this service, or what it is in their practices that makes uptake lower? In the conclusions you say that adopters differ systematically, but I can’t read out from your paper anything about the why, are for example some providers more exposed to the barriers you have identified?

Thank you for this comment. Our research group had two companion papers to further investigate the pattern of utilization (Luo et al. 2021) and the reasons for utilization (Nederveld et al. 2021), thus we did not speculate too much in this manuscript. We did theorize why we think utilization was low in general based on barriers as well as potential solutions in Table 2 (which was missing from the original paper and has now been included and renumbered as Table 3 in the updated draft). However, we agree with the reviewer a brief discussion about how this can be used to improve utilization would be beneficial. We added some discussions of the patterns on p.10:

“Specifically, being female, non-internal medicine PCP, practicing in the Midwest region and small clinics were associated with lower probabilities of being an IBT user. Having higher proportions of patients with hyperlipidemia was associated with higher probabilities of being a late adopter, an early adopter, or an always user. This relationship between the patient composition and provider IBT-uptake was consistent with the need for obesity management in patients with high lipids. These patterns were consistent with practice-level characteristics associated with IBT uptake (e.g., larger practices are more likely to use IBT) [18], geographic distribution of the Medicare obese population (e.g., prevalence of obesity is highest in the South) [19], and the complexity of obesity management (e.g., some PCPs such as nurse practitioners need more formal obesity treatment training) [20].”

Look through the methods section, in particular the Data analysis-section, and make sure all conducted analyses are presented here. It is now focusing on the data, not the analyses presented in the Results section. Moreover, I believe your paper would benefit from adding some more statistical analyses, such as comparing proportions between groups and variation in the number of beneficiaries per provider (mean and confidence intervals for example). Even regressions to identify to joint effect of different characteristics on use could be considered. Now it’s very descriptive, just really presenting the aggregated data without in depth analysis.

We added the statement of all analyses performed. Because this paper was mainly descriptive, we did not perform statistical testing. However, in accordance with the comment we added regression analyses. For the four IBT provider groups (never user; late adopters; early adopters; always users) we used a multinomial logistic regression to find statistically significant factors in Table 1 related to the groups. In the Method section we added this statement on p.6-7:

“Descriptive statistics (sample size, proportions for discrete variables, median, means and standard deviations for continuous variables) were presented by provider user types. Multinomial logistic regressions were used to compare provider characteristics and patient-compositions related to utilization patterns. Two-sided tests at the 0.05 significance level were performed using Stata 17 (StataCorp LLC).”

I believe readers would have use of a more thorough description of the intervention/service you are studying. Of course, it’s possible to go to other sources for details, but now it’s only very briefly described. Who is this service for, who is eligible, what does it include, how is it provided?

We added more description of the service in the Background section on p.3:

“IBT consists of working closely with an approved provider to help target poor habits that are contributing to a patient’s obesity condition and helping educate the patient and implement changes such as, track eating and creating an exercise plan. All Medicare obese beneficiaries are eligible for the service [10]. The benefit allows for 15-min visits once per week for 4 weeks, biweekly visits for months 2 to 6, and then once monthly for another 6 months if the patient lost ≥ 3 kg (6.6 lb) [11].”

MINOR COMMENTS
Overall, the text suffers from many abbreviations. Would it be possible to reduce?

We deleted the abbreviation POSPUF, NPI and RD from the text and replaced them by simpler and unambiguous terms.

Background:
For me it was a bit unclear what “B” rating and B deductible is, and maybe for many other non-US readers.

We added what the “B” rating means and the corresponding reference on p.3:

“IBT is an evidence-based service with a “B” rating, meaning there is “high certainty that the net benefit is moderate or there is moderate certainty that the net benefit is moderate to substantial, and is recommended by the United States Preventive Services Task Force to be provided by clinicians to eligible patients [8].”

The “B deductible” in the original text was “Medicare Part B deductible.” To avoid confusion, it is changed to “Medicare Part-B deductible” on p.3.

What do you mean by “reassessed”, I could not see from the text that beneficiaries were ever assessed in the first place?

To be eligible for the service the beneficiary needs to be assessed before IBT was provided. To be eligible, a Medicare beneficiary has to have BMI ≥ 30 kg/m^2^. This is now made clearer on p.4:

“If the patient does not achieve the weight loss goal, they must wait at least 6 months and be assessed for eligibility based on BMI in order to begin a new treatment period once again.”

You say the uptake “has been underwhelming”, but how bad and is there a reference for that?

We added references and clarified the statement as follows on p.4:

“The uptake of IBT services among Medicare providers has been underwhelming (0.1% of eligible beneficiaries in 2012 [13] and 0.2% in 2015 [14]) since the CMS authorized billing in 2011.”

Methods:
So seldom-users are excluded, I guess this means that it is possible that some provider characteristics in the groups had changed if you included all users, and that e.g., some late adopters could even be always users? Now you use an estimate of 5000 seldom users, but without reference or argument for it. Can this be clarified in the methods and also a bit more discussed in the limitations section? Even an analysis showing how use is distributed by characteristic could provide some more information to this issue, is there for example provider groups that are very close to being listed as non-users because of this limit of 10 beneficiaries?

The Medicare publicly available data exclude providers with less than 10 beneficiaries for this service (and all service codes), so we do not have access to the data and are not able to include this data in the analysis. This was included in the original text as a limitation. The reference to 5,000 seldom users was just our best guestimate. From the characteristics of never users and other adopters, we cannot find any algorithm to identify seldom users. Thus, we eliminated the referent to 5,000 seldom users in the revised manuscript. In the Method section, we clarified:

“To protect the privacy of Medicare beneficiaries, any aggregated records that are derived from 10 or fewer beneficiaries are excluded from the public use data, which led to some providers being classified as a non-user even though they had used IBT for a handful of patients.” (p.5)

In the Limitation section we added:

“One limitation of this work is that we were not able to assess providers who did provide IBT for obesity but only for 10 or fewer beneficiaries each year because of data suppression in the CMS publicly available data, which may lead to a slight underestimate. In addition, some late adopters may have been misclassified when they used some IBT services in the early years to a lesser extent.” (p.12)

What is the study design, should I view this as an ecological study or how linked are the data sources?

This is a cohort study of five types of primary care providers who were authorized to provide IBT services and who billed Medicare for more than 10 unique beneficiaries for any service. Providers were “followed-up” i.e., linked, over time using their unique national provider identifier. The unit of analysis was providers. The provider’s listed practice state was linked to America’s Health Ranking’s obesity data and Kaiser Family Foundation’s publications are used to find obese Medicare beneficiary populations by state. There was no other linkage of data sources. We clarified by adding this statement in the Method section on p.5:

“We conducted a cohort analysis of primary care providers who were authorized to provide IBT services and who billed Medicare for more than ten unique beneficiaries for any service between 2013 and 2020 to examine their IBT service uptake patterns.”

Results:
Could you discuss your results that chronic conditions were similar regardless IBT-uptake, how should I interpret this?

This was a descriptive statement about the proportions of patients having chronic conditions across the IBT-uptake group. However, not all differences were statistically significant. In the revised manuscript we used a multinomial model to jointly test provider characteristics and patient compositions in relation to IBT-uptake patterns. The new Table 2 shows that compared to never users, for later adopters the odds ratios (ORs) for every 10 percentage points increase in patients with hypertension, diabetes, and hyperlipidemia are 0.94 (95% CI 0.90, 0.98), 0.92 (0.90, 0.95), and 1.26 (1.22, 1.30), respectively. For early adopters the corresponding ORs are 0.96 (0.87, 1.05), 0.86 (0.82, 0.91), and 1.49 (1.40, 1.58), respectively; and finally for always users 0.78 (0.61, 1.01), 1.07 (0.95, 1.21), and 1.81 (1.52, 2.16), respectively. These suggested that controlling for provider gender, region, type, and size of practices, having higher proportions of patients with hypertension was associated with lower probabilities of being a late adopter, having higher proportions of patients with diabetes was associated with lower probabilities of being a later adopter and an early adopter, and having higher proportions of patients with hyperlipidemia was associated with higher probabilities of being a late adopter, an early adopter or an always user. This relationship between patient compositions and provider IBT-uptake is consistent with the need for obesity management in patients with high lipids.

We now added these sentences in the Results section on p.8-9:

“Table 2 showed the odds ratio (OR) of a multinomial logistic regression with the four uptake patterns as the outcome and factors in Table 1 as regressors (excluding the highly collinear number of annual billed HCPCS codes). The provider types other than internal medicine were grouped together to avoid the small cell problem. The 95% confidence intervals (CIs) indicated that male, Northeast- and South-region, and internal medicine providers had an increasing likelihood of being a late, early, or always adopters compared to never users. Compared with never users, for every 10 percentage points increase in patients with hypertension, diabetes, and hyperlipidemia among later adopters, the ORs were 0.94 (95% CI 0.90, 0.98), 0.92 (0.90, 0.95), and 1.26 (1.22, 1.30), respectively. For early adopters vs. never users the corresponding ORs were 0.96 (0.87, 1.05), 0.86 (0.82, 0.91), and 1.49 (1.40, 1.58), respectively; and finally for always users vs. never users 0.78 (0.61, 1.01), 1.07 (0.95, 1.21), and 1.81 (1.52, 2.16), respectively.”

Just to be clear I’m reading this correct, the numbers in parentheses that reports the number of beneficiaries per 1000 patients with obesity are e.g., 27.5 of 1000, and not percentages, so even in the areas with highest uptake its less than 3% of beneficiaries?

That is current.

Discussion:
You write about low reimbursement rates. How low is it, compared to alternative uses of the same amount of time by these providers? If this is not prioritized, what is and why?

The rate of reimbursement for a 15-min consultation of IBT is $26, whereas payment for a typical evaluation and management code for an established patient for a level-2 visit (CPT code 99212) is $45 and a level-3 visit (CPT code 99213) $74 (Medicare payment for calendar year 2018). Thus, a provider may receive higher reimbursement for similar services using the other codes. From the reimbursement rates we may infer that obesity related IBT was not prioritized. We added this Discussion on p.11:

“The rate of reimbursement for a 15-min consultation of IBT is $26, whereas payment for a typical evaluation and management code for an established patient for a level-2 visit (CPT code 99212) is $45 and a level-3 visit $74 (CPT code 99213, Medicare payment for calendar year 2018). While CMS aimed to increase utilization of preventive service and screenings through procedure codes, the uptake rates of these services have been low. For example, 3% of Medicare FFS beneficiaries received a visit specifically addressing depression screening in 2016 [22]. The G-codes for Annual Wellness Visits were introduced in 2011 with much higher allowable charge than obesity counseling services but the penetration was only 16~17% in 2014 [23,24].”

What do you mean by “these requirements may be difficult for patients”, what is requirements here?

This was referring to the expectation of the patient to be completing weekly visits for the first month and twice a month for the next five months. We have clarified the language on p.11:

“In addition, these frequent visits may be difficult for patients to achieve in certain populations with other comorbid conditions such as diabetes and patients with more resource constraints such as transportation barriers.”

What do you mean by “under their direct supervision”? Would it for example be enough to have a dietician be employed by one of the eligible providers?

As far as we know it is not enough to have a dietician employed by an eligible provider to independently provide IBT. Referral to RDs and other auxiliary providers who work outside of the primary care setting is not covered by CMS. We have clarified the language on p.11:

“A non-physician auxiliary practitioner, such as a registered dietician, may provide IBT if they must bill “incident to” the primary care physician, who must be physically present at the time services are provided. Referral to RDs and other auxiliary providers who work outside of the primary care setting is not covered by CMS [11].”

You present numbers within parentheses (e.g., “46,821 versus 44,572”) but its difficult to see which number is which, could you add years in the parentheses also?

The numbers in the parentheses are not representing different years, they are representing the same years, but comparing our data to another source measuring similar variables, the paper has been updated to clarify this point. We have updated the language on p.12-13:

“Compared with the previous report [14] our estimated number of beneficiaries in 2013 was slightly lower (46,821 from the previous report versus our finding of 44,572). Therefore, our results may underrepresent the true amount of IBT for obesity happening in the earlier years. However, our estimates in 2014 (46,171 from the previous report versus our finding of 57,488) and 2015 (57,576 from the previous report versus our finding of 73,234) were higher which might be due to the updates of final-action claim items over time.”

How many providers are expected to be among the “not in the National plan” until after 2014, or deactivated during the study period?

Using the 2013-2022 data, we found that out of the 575,936 PCPs in our cohort, 239,919 did not have more than 10 unique fee-for-service Medicare beneficiaries until after 2014. However, this cannot be considered new Medicare providers because majority of them might have served Medicare patients but due to privacy considerations the public use data suppressed that information. Had we limited our cohort to those who served at least 11 Medicare patients in 2013 or 2014, the number of never users and later adopters would reduce to 330,109 and 4,315 (vs. 568,941 and 5,391 in the current analysis) and the numbers of early adopters and always users remain the same.

Using the CMS NPPES (National Plan and Provider Enumeration System) data, we found 141,313 out of the 575,936 PCPs in our cohort had the first enumeration year after 2014. Among these 140,808 were classified as never users in our analysis and 505 as late adopters. Using the CMS Deactivated Providers list as of July 2022, we found 6,049 of the 575,936 PCPs in our cohort were deactivated in the study period, among whom 5,985 were never users, 35 were late adopters and 29 were early adopters.

We clarified these points on p.13:

“A third limitation of the study is that when we classified providers into early, late, always or never adopters we did not exclude providers who were not in the National Plan and Provider Enumeration System until after 2014 or providers who were deactivated between 2013 and 2020. We found 141,313 out of the 575,936 PCPs in our cohort had the first enumeration year after 2014, among whom 140,808 were classified as never users in our analysis and 505 as late adopters. Using the CMS Deactivated Providers list as of July 2022, we found 6,049 of the 575,936 PCPs in our cohort were deactivated in the study period, among whom 5,985 were never users, 35 were late adopters and 29 were early adopters. We do not expect these limitations to affect our results qualitatively.”

Reviewer #2: The manuscript presents updated data on the usage of the CMS IBT benefit by primary care providers between 2013 and 2019. Updated data and information about trends over time in the adoption of the CMS IBT benefit are useful and the inclusion of provider characteristics provides novel information not included in previous reports of CMS IBT uptake. I would recommend revisions that would make the adopter subgroups more useful to the reader as well as allow more definitive conclusions about the differences between provider subgroups.

Thank you for your comment. At the time of revision, we found the 2020 data for the Medicare Fee-for-service Provider Utilization and Payment data for Physician and Other Supplier Public Use File as well as the Provider Summary Tables from CMS became available. To provide more up to date information, we took the opportunity to add the year 2020 to the manuscript.

• Please use person first language throughout the manuscript.

We believe the paper is currently in first person language, please provide clarification for this request.

• The tables are missing from the manuscript.

Tables were uploaded separately per PLOS ONE requirements but have now been added back into the paper.

• I found the early adopters vs late adopter categories to be less useful because they do not provide much information about the frequency with which providers that only occasionally reached the threshold of 10 claims per year were using the IBT codes. For example, some of the “early adopters” might have continued to use IBT services for at least 10 patients in most years, while others may have tapered off completely. Other data provided in the Figures already captures more meaningful information about changes in provider usage over time. Perhaps a different division such as “rare users” who had 10 claims in <50% of years vs “frequent users” >50% of years would provide more meaningful information about usage patterns. The “always user” category could be maintained though it is quite small. Alternatively, the authors could simply present data showing the percent of providers who met the threshold of 10 beneficiaries in only 1 yr, in 2-3 of the yrs, 4-5 yrs, etc.

Following the grouping of providers who met the threshold of 10 beneficiaries in <50% of years, i.e., “rare users” (1-4 years between 2013-2020), “frequent users” (5-7 years). and always users (8 years), we found the counts and percentages are close to the original groups (see Table R1 below).

| **Table R1.** | **Never** | **Rare** | **Frequent** | **Always** |
| --- | --- | --- | --- | --- |
|  | N=568941 | N=5921 | N=867 | N=207 |
| Gender |  |  |  |  |
| F | 352888 (62.0%) | 2586 (43.7%) | 317 (36.6%) | 61 (29.5%) |
| M | 216053 (38.0%) | 3335 (56.3%) | 550 (63.4%) | 146 (70.5%) |
| Region |  |  |  |  |
| MIDWEST | 136789 (24.0%) | 894 (15.1%) | 106 (12.2%) | 22 (10.6%) |
| NORTHEAST | 116862 (20.5%) | 1418 (23.9%) | 270 (31.1%) | 73 (35.3%) |
| SOUTH | 207676 (36.5%) | 2573 (43.5%) | 366 (42.2%) | 85 (41.1%) |
| WEST | 107614 (18.9%) | 1036 (17.5%) | 125 (14.4%) | 27 (13.0%) |
| Provider type |  |  |  |  |
| Family Practice | 111065 (19.5%) | 2123 (35.9%) | 341 (39.3%) | 67 (32.4%) |
| General Practice | 7628 (1.3%) | 134 (2.3%) | 19 (2.2%) | 2 (1.0%) |
| Internal Medicine | 136999 (24.1%) | 2526 (42.7%) | 466 (53.7%) | 137 (66.2%) |
| Nurse Practitioner | 201314 (35.4%) | 870 (14.7%) | 29 (3.3%) | 1 (0.5%) |
| Physician Assistant | 111935 (19.7%) | 268 (4.5%) | 12 (1.4%) | 0 (0.0%) |
| Median annual number of unique Medicare beneficiaries | 102 | 261 | 341 | 356 |
| Median annual number of services/encounters per year | 247 | 1636 | 2930 | 4349 |
| Median annual number of all procedure codes | 16 | 43 | 55 | 66 |
| Median annual submitted charge amount | 41530 | 167028 | 297368 | 367496 |
| Percent of beneficiaries with hypertension | 68.1 (9.6) | 69.8 (7.7) | 70.2 (7.2) | 71.6 (6.1) |
| Percent of beneficiaries with diabetes | 38.4 (13.3) | 38.7 (13.4) | 39.4 (14.4) | 43.0 (16.0) |
| Percent of beneficiaries with hyperlipidemia | 57.4 (13.0) | 61.1 (12.1) | 63.6 (11.7) | 65.8 (10.8) |

Alternatively, using providers who met the threshold of 10 beneficiaries in only 1 yr, in 2-3 of the yrs, 4-5 yrs, 6-7 yrs, and 8 years, the results are also similar (see Table R2 below).

In both regroupings, the counts and percentage are very close to the original classification. However, using the different groupings suggested in Tables R1 and R2 would be answering a somewhat different question, more about frequency of utilization, whereas the main aim of this paper is to understand how utilization has changed over the years starting from the year the service was introduced, in order to understand if providers were more likely to provide the service right away, later or on, and if they continued to provide the service throughout the years. We would be happy to provide these groupings in supplemental materials if needed.

| **Table R2** | **Never** | **1 yr** | **2-3 yrs** | **4-5 yrs** | **6-7 yrs** | **8 yrs** |
| --- | --- | --- | --- | --- | --- | --- |
|  | N=568941 | N=2795 | N=2528 | N=1008 | N=457 | N=207 |
| Gender |  |  |  |  |  |  |
| Female | 352888 (62.0%) | 1292 (46.2%) | 1068 (42.2%) | 378 (37.5%) | 165 (36.1%) | 61 (29.5%) |
| Male | 216053 (38.0%) | 1503 (53.8%) | 1460 (57.8%) | 630 (62.5%) | 292 (63.9%) | 146 (70.5%) |
| Region |  |  |  |  |  |  |
| MIDWEST | 136789 (24.0%) | 452 (16.2%) | 350 (13.8%) | 152 (15.1%) | 46 (10.1%) | 22 (10.6%) |
| NORTHEAST | 116862 (20.5%) | 630 (22.5%) | 640 (25.3%) | 267 (26.5%) | 151 (33.0%) | 73 (35.3%) |
| SOUTH | 207676 (36.5%) | 1216 (43.5%) | 1115 (44.1%) | 417 (41.4%) | 191 (41.8%) | 85 (41.1%) |
| WEST | 107614 (18.9%) | 497 (17.8%) | 423 (16.7%) | 172 (17.1%) | 69 (15.1%) | 27 (13.0%) |
| Provider type |  |  |  |  |  |  |
| Family Practice | 111065 (19.5%) | 985 (35.2%) | 890 (35.2%) | 414 (41.1%) | 175 (38.3%) | 67 (32.4%) |
| General Practice | 7628 (1.3%) | 62 (2.2%) | 61 (2.4%) | 18 (1.8%) | 12 (2.6%) | 2 (1.0%) |
| Internal Medicine | 136999 (24.1%) | 1091 (39.0%) | 1155 (45.7%) | 492 (48.8%) | 254 (55.6%) | 137 (66.2%) |
| Nurse Practitioner | 201314 (35.4%) | 509 (18.2%) | 314 (12.4%) | 65 (6.4%) | 11 (2.4%) | 1 (0.5%) |
| Physician Assistant | 111935 (19.7%) | 148 (5.3%) | 108 (4.3%) | 19 (1.9%) | 5 (1.1%) | 0 (0.0%) |
| Median annual number of unique Medicare beneficiaries | 102 | 237 | 274 | 321 | 346 | 356 |
| Median annual number of services/encounters per year | 247 | 1360 | 1746 | 2625 | 3159 | 4349 |
| Median annual number of all procedure codes | 16 | 40 | 44 | 53 | 57 | 66 |
| Median annual submitted charge amount | 41530 | 141772 | 185130 | 255970 | 306295 | 367496 |
| Percent of beneficiaries with hypertension | 68.1 (9.6) | 69.6 (7.9) | 69.9 (7.4) | 69.9 (7.7) | 70.6 (6.7) | 71.6 (6.1) |
| Percent of beneficiaries with diabetes | 38.4 (13.3) | 38.6 (13.3) | 39.0 (13.4) | 38.5 (14.0) | 40.3 (14.4) | 43.0 (16.0) |
| Percent of beneficiaries with hyperlipidemia | 57.4 (13.0) | 60.6 (12.5) | 61.5 (11.7) | 62.1 (12.3) | 64.1 (11.2) | 65.8 (10.8) |

• Although I cannot fully review this information without the tables, it appears likely that the authors are making claims about differences between provider usage groups when no statistical comparisons were conducted.

Following Reviewer 1’s comment we estimated a multinomial logistic regression to find statistically significant differences between groups. A new Table 2 was added, and the Results and Discussion sections were updated accordingly.
