# Supplementary material for: Primary care provider uptake of intensive behavioral therapy for obesity in Medicare patients, 2013–2019
Source: PLoS One. 2023 Mar 24;18(3):e0266217. doi: 10.1371/journal.pone.0266217 (PMC10038247; doi:10.1371/journal.pone.0266217)
Supplement: Attachment — Submitted filename: Rebuttal.docx [file pone.0266217.s004.docx]

Dear Editor,

Thank you for the helpful comments by the academic editor and reviewers and for the opportunity to revise the manuscript. Below we address each reviewer comment. All page numbers referenced in this letter are those of the revised manuscript with track change. The blue font type is for our response and the brown font type is for the revised text.

Dear Author,

Thank you very much for this quick answer

Our reviewers ended the second review and still some minor changes are thought to be addressed.

Please pay attention to those from reviewer 2 and some of the language, rewording and rephrasing recommendations.

Response: We thank the Editor for giving us the opportunity to revise our manuscript. We believe we have addressed all comments below.

Reviewer #1: Thank you for your careful revisions. I have no further comments and also learnt a couple of things from reading it, both about the Medicare services and the distinction between cohort analysis (a term I must admit I had not heard before, despite my background in register-based research) and cohort study.

Response: Thank you for the feedback.

Reviewer #2: I thank the authors for their revisions, which have improved and clarified the manuscript.

Person first language refers to separating the person from words that describe a medical or mental health condition of that person. This would mean changing all instances of “obese people/individuals/beneficiaries etc.” to “people/individuals/beneficiaries with obesity” and changing statements like “x% of the population are obese” to “x% of the population have obesity”. This format is now a standard requirement in most research publications. Please modify your language throughout the abstract and manuscript; they do not currently use person first language. I would also avoid using terms like “poor habits” which imply a value judgment and instead just use “habits (or behaviors) that contribute to the patient’s obesity (p. 3 of track changes text).

Response: Thank you for the explanation. We changed all instances of this issue to person first language throughout the manuscript and the abstract. A total of 13 occurrences were corrected.

I actually would recommend against the re-calculation of the usage pattern groups based on the added 2020 data. The inclusion of 2020 further reduced the size of the “always user” group. As the authors note regarding their decision not to graph utilization for 2020 (p. 6), the utilization in that year was likely lower than previous years due to the pandemic. The IBT benefit requires patients to attend in person visits, and it is unlikely that many medical providers would have recommended that patients with obesity (who were at high risk of COVID-19 complications) attend in-person elective counseling early in the pandemic. There also were likely regional differences in providers’ and patients’ willingness to attend in-person care during that time. Thus the overall results are now being influenced by data from an atypical year. The authors instead might describe usage in 2020 separately, if desired, or revert to the previous time period.

Response: Thank you for this recommendation. We agree and revert the analysis to the previous time period. We added a separate paragraph in the Discussion section to present the 2020 data and explanation for the exclusion (p.12):

“At the time of the study, the 2020 data became available. However, we decided to exclude it from our analyses because the data may be misleading due to the effect on health care visits of the early part of the COVID-19 pandemic. The number of claims dropped to 245,442 (a 15% drop compared with 2019 data) and the number of unique beneficiaries dropped to 134,560 (a 11% drop). Access to non-emergent health care services was limited during the last months of 2020.”

I also do not understand how the number of early adopters could change by ~300 given that that category was defined by adoption in either 2013 or 2014 and therefore should not have been influenced by the addition of 2020 data (except for by the re-categorization of the 32 people originally classified as “always users”). Was there an error in the original analysis? Similarly why did the number of late adopters increase by 1000? Were all of those adopters in 2020?

Response: We apologize for not explaining this in the previous response letter. There were two reasons why the numbers changes. One was as the reviewer pointed out 32 “always users” became “early adopters,” and the number of “late adopters” increased by those who started the service in 2020. The second reason was a programming error in the original analysis. Providers who provided both the individual and the group services were inadvertently excluded in the counts, that is, only providers who billed for individual treatment or who billed for group treatment were included, not both. This will explain why when we revert to the 2013-2019 analysis in this revision, the numbers are not the same as either of the previous versions. In this version, the numbers differ from the original submission because of the second reason. Only one state’s heat map was affected, which was the providers in 2019.

I do think that the manuscript would benefit from the addition of the provider usage frequency information to the supplement as a complement to the data presented in the primary manuscript.

Response: Thank you for the recommendation. We added an online supplement to provide the provider usage frequency information. For your convenience, the supplement is included in the end of this letter.

Please remove statements such as “indicating that the increase in the prevalence of these chronic conditions in the populations that the providers served did slightly impact whether or not they provided IBT obesity services to their patients overall” and any other causal statements that are based on descriptive differences that did not achieve statistical significance (and could not determine causality to begin with). Note that some of these ORs were actually significant in the opposite direction (% pts with diabetes was associated with 14% lower odds of adoption). Odds ratios for region are only interpretable in comparison to the reference category (Midwest).

Response: Thank you for this comment. We change the aforementioned statement to (p.8):

“The proportions of patients with these chronic conditions were fairly similar but did slightly increase across the four IBT-uptake groups (lower panel Table 1), showing somewhat of a pattern between the prevalence of the relative chronic conditions in the populations that the providers served and whether or not they provided IBT obesity services to their patients.”

We also examined throughout the manuscript and attempted to revise any similar statements. For example, we changed “improvement” to “increase” on p.9:

“The state that saw the largest increase in the absolute number of IBT providers per 1,000 providers was Nevada (0 to 18.3 from 2013 to 2019).”

The phrasing of this statement is confusing, please reword (and I would recommend changing in favor of a description of the results since the ORs are listed in the table): “Compared with never users, for every 10 percentage points increase in patients with hypertension, diabetes, and hyperlipidemia among later adopters, the ORs were 0.94 (95% CI 0.90, 0.98), 0.92 (0.90, 0.95), and 1.26 (1.22, 1.30), respectively.”

Response: We apologize for the confusion. The sentence was revised to read (p.8):

“Compared with never users, for every 10 percentage points increase in patients with hypertension among later adopters, the OR for being a late adopter was 0.92 (95% CI 0.90, 0.96). Similarly, for every 10 percentage points increases in patients with diabetes, the OR for being a late adopter was 0.93 (95% CI 0.90, 0.96) and the OR for being an early adopter was 0.87 (95% CI 0.82, 0.91). However, for every 10 percentage points increases in patients with hyperlipidemia, the OR for being a late adopter was 1.28 (95% CI 1.24, 1.32), the OR for being an early adopter was 1.50 (95% CI 1.41, 1.60), and the OR for being an always user was 1.92 (95% CI 1.63, 2.26). “

For Table 2 what was the percentage of missing data for the marked outcomes?

Response: There is no missing data for the outcome. Missing values were for the covariates. The percentages of missing data for hypertension, diabetes, and hyperlipidemia were 6.7%, 18.7%, and 11.2%. This information is added to the footnote of Table 2.

**Supplemental materials**

In addition to classifying providers as we did in the manuscript (early, late, always and never adopters), we also summarize the IBT uptake patterns among eligible primary care providers based on the total number of years they served at least 10 Medicare beneficiaries. A provider is a “rare user” if they used IBT for <50% of the years (1-3 years) or a “frequent user” if they used IBT for >50% (4-7 years). Table S1 shows the provider characteristics by this classification and Table S2 shows the multinomial logistic regression estimates.

The characteristics of providers who rarely used IBT (1-3 years out of 7 years) were similar to the late adopters in Table 1 and the frequent users (4-7 years) combined the characteristics of early adopters and always users.

The odds ratios (ORs) of the multinomial logistic regression with the three uptake categories in Table S2 were similar to those in Table 2. Again, the ORs for rare users were almost the same as the later adopters and the ORs for frequent users were in between the ORs for early adopters and always users (except for the Southwest region).

**Table S1** Provider characteristics by intensive behavioral therapy (IBT) uptake patterns in three categories (N=575,936) from 2013 to 2020

|  | **Never** | **Rare (1-3 years)** | **Frequent (4-7 years)** |
| --- | --- | --- | --- |
|  | N=531,587 | N=4,851 | N=1,316 |
| Gender |  |  |  |
| Male | 206528 (38.9%) | 2749 (56.7%) | 837 (63.6%) |
| Female | 325059 (61.1%) | 2102 (43.3%) | 479 (36.4%) |
| Region |  |  |  |
| Midwest | 128375 (24.1%) | 742 (15.3%) | 164 (12.5%) |
| Northeast | 109266 (20.6%) | 1186 (24.4%) | 401 (30.5%) |
| South | 193229 (36.3%) | 2084 (43.0%) | 567 (43.1%) |
| West | 100717 (18.9%) | 839 (17.3%) | 184 (14.0%) |
| Provider Type |  |  |  |
| Family Practice | 107270 (20.2%) | 1751 (36.1%) | 504 (38.3%) |
| General Practice | 7428 (1.4%) | 118 (2.4%) | 26 (2.0%) |
| Internal Medicine | 132094 (24.8%) | 2110 (43.5%) | 715 (54.3%) |
| Nurse Practitioner | 180882 (34.0%) | 651 (13.4%) | 55 (4.2%) |
| Physician Assistant | 103913 (19.5%) | 221 (4.6%) | 16 (1.2%) |
| Median annual number of unique Medicare beneficiaries | 108 | 268 | 344 |
| Median annual number of services/encounters per year | 264 | 1690 | 3096 |
| Median annual number of all procedure codes | 16 | 44 | 58 |
| Median annual submitted charge amount | 43955 | 173387 | 296086 |
| Percent of beneficiaries with obesity related chronic conditions ‡ | | | |
| Hypertension | 68.0% | 69.7% | 70.4% |
| Diabetes | 38.2% | 38.8% | 39.8% |
| Hyperlipidemia | 57.1% | 61.0% | 63.6% |

‡ Percentages based on providers with available data from Provider Summary Tables.

**Table S2** **Multinomial logistic regression of IBT update patterns in three categories and provider characteristics and patient composition.**

|  | **Never** | **Rare （1-3 years）** | | **Frequent (4-7 years)** | |
| --- | --- | --- | --- | --- | --- |
|  |  | **OR** | **(95% CI)** | **OR** | **(95% CI)** |
| Male | 38.9% | Ref |  | Ref |  |
| Female | 61.1% | 0.68 | (0.64,0.72) | 0.58 | (0.51,0.65) |
| Midwest | 24.1% | Ref |  | Ref |  |
| Northeast | 20.6% | 1.69 | (1.54,1.86) | 2.35 | (1.95,2.82) |
| South | 36.3% | 1.67 | (1.54,1.82) | 1.95 | (1.63,2.32) |
| West | 18.9% | 1.49 | (1.35,1.65) | 1.49 | (1.21,1.84) |
| Other PCPs† | 75.2% | Ref |  | Ref |  |
| Internal medicine | 24.8% | 1.54 | (1.44,1.64) | 2.06 | (1.82,2.32) |
| # Medicare beneficiaries /100 | 1.8 | 1.08 | (1.07,1.09) | 1.08 | (1.07,1.09) |
| # services encounters /100 | 9.4 | 1.00 | (1.00,1.00) | 1.00 | (1.00,1.00) |
| $ submitted charges /10,000 | 11.2 | 1.04 | (1.03,1.04) | 1.05 | (1.04,1.05) |
| % patients w/ hypertension /10 ‡ | 6.8 | 0.94 | (0.90,0.99) | 0.81 | (0.74,0.89) |
| % patients w/ diabetes /10 ‡ | 3.8 | 0.92 | (0.90,0.95) | 0.92 | (0.88,0.97) |
| % patients w/ hyperlipidemia patients /10 ‡ | 5.7 | 1.28 | (1.24,1.32) | 1.62 | (1.51,1.72) |

† Other PCPs include family practice, general practice, nurse practitioner, and physician assistant.

‡ Missing data were imputed by the mean value. The percentages of missing data for hypertension, diabetes, and hyperlipidemia were 6.7%, 18.7%, and 11.2%.
